# Two novel missense mutations in the myelin protein zero gene causes Charcot-Marie-Tooth type 2 and Déjérine-Sottas syndrome

**DOI:** 10.1186/1756-0500-3-99

**Published:** 2010-04-12

**Authors:** Geir J Braathen, Jette C Sand, Michael B Russell

**Affiliations:** 1 Faculty Division Akershus University Hospital, University of Oslo, 1474 Nordbyhagen, Oslo, Norway; 2 Head and Neck Research Group, Research Centre, Akershus University Hospital, 1478 Lørenskog, Oslo, Norway

## Abstract

**Background:**

The Charcot-Marie-Tooth (CMT) phenotype caused by mutation in the *myelin protein zero (MPZ) *gene varies considerably, from early onset and severe forms to late onset and milder forms. The mechanism is not well understood. The myelin protein zero (P_0_) mediates adhesion in the spiral wraps of the Schwann cell's myelin sheath. The crystalline structure of the extracellular domain of the myelin protein zero (P_0_ex) is known, while the transmembrane and intracellular structure is unknown.

**Findings:**

One novel missense mutation caused a milder late onset CMT type 2, while the second missense mutation caused a severe early onset phenotype compatible with Déjérine-Sottas syndrome.

**Conclusions:**

The phenotypic variation caused by different missense mutations in the *MPZ *gene is likely caused by different conformational changes of the MPZ protein which affects the functional tetramers. Severe changes of the MPZ protein cause dysfunctional tetramers and predominantly uncompacted myelin, i.e. the severe phenotypes congenital hypomyelinating neuropathy and Déjérine-Sottas syndrome, while milder changes cause the phenotypes CMT type 1 and 2.

## Background

Charcot-Marie-Tooth (CMT) disease is characterized by distal muscle wasting and weakness, sensory loss with reduced tendon reflexes and foot deformities [1,2]. It is the most common inherited disorder of the peripheral nervous system with an estimated prevalence of 1 in 2,500 [3]. CMT is a heterogeneous disorder with respect to clinical features, neurophysiology, pathophysiology and genetics. The number of identified CMT genes is still expanding. So far the majority of the CMT genes encodes either neuronal or Schwann cell proteins [4]. However, the most frequent autosomal recessive form of CMT is caused by mutation in the glioside-induced differentiation-associated protein-1 (*GDAP1*) gene, which is located in the mitochondrial outer membrane and expressed in both neurons and Schwann cells [5].

The myelin protein zero (*MPZ*) gene encodes a leader peptide of 29 amino acids which is cleaved from the remaining 219 amino acids before insertion in the myelin sheath [6]. The myelin protein zero (P_0_) is post-translationally modified by additions of multiple sulphate, acyl and phosphate groups and an N-linked oligosaccharide [7,8]. P_0 _has an extracellular, a transmembrane and a cytoplasmic domain. It is the most abundant protein in the myelin sheath of the peripheral nervous system [9]. The extracellular domain from myelin protein zero protein (P_0_ex) mediates homotypic adhesion, and it is essential for the formation of the compact myelin layer of Schwann cells [10,11]. It is included in the immunoglobulin supergene family, all members of which are involved in recognition and/or adhesion. X-ray crystallography suggests that P_0_ex emanate from the membrane surface as tetramers that link to tetramers on the opposing membrane surface, to result in the formation of networks of molecules [12]. Knock-out mice without P_0 _expression develop a severe dysmyelinating neuropathy with predominantly uncompacted myelin [13].

The paper describes two novel missense mutations in the *MPZ *gene, and discusses possible mechanism in relation to the phenotypic variability caused by different missense mutations in the *MPZ *gene.

## Methods

### Patients

Norwegian patients with CMT disease were analyzed for mutations in the *MPZ *gene. Affected were interviewed and had a neurological examination. The Neuropathy Impairment Score (NIS) and Charcot-Marie-Tooth disease neuropathy score (CMTNS) were used [14,15]. Nerve conduction velocities were recorded with surface electrodes, while needles were used for electromyography (EMG).

### Genetics

DNA was extracted from leucocytes using QIAgen FlexiGene kit (Düsseldorf, Germany). The sequences were derived from the National Center for Biotechnology Information (NP_000521 and NM_000530, *MPZ*). The coding region of the *MPZ *gene as well as exon and intron junctions were amplified using Eppendorf hotmaster taq polymerase. The sequencing was carried out using forward, reverse and internal primers, and BigDye Terminator kit version 1.1 Applied Biosystems (Life Technologies, Carslbad, CA, USA). Sequencing was performed using Applied Biosystems ABI3130x1 Genetic analyzer and aligned with the Sequencher programme (Gene Codes Corporation, Ann Arbor, MI). Numbering nomenclature used for the mutations includes the leader peptide of 29 amino acids.

### Statistical analysis

SPSS for Windows version 14.0 was used for statistical analysis.

### Ethics

The study was approved by the Regional Committees for Medical Research Ethics.

## Results

Table 1 shows the demographics, clinical characteristics, phenotypes and novel *MPZ *missense mutations in three families. Additional file 1, table S2 shows the neurophysiology in the three families. The affected from family 1 was an immigrant from the Philippines, while family 2 and 3 originated from the same county and carried the same missense mutation. The affected in family 1 had early onset and severe symptoms compatible with Déjérine-Sottas syndrome (DSS), while affected in family 2 and 3 had late onset milder symptoms and axonal neuropathy compatible with CMT type 2.

**Table 1:** Demographics, clinical characteristics, neurophysiology, phenotype and missense mutation for the three families.

Family	1	2		3
			
Family member		Father	Daughter	
*Demographic*				
Sex	♀	♂	♀	♂
Age at onset	2	70	29	56
Disease duration	13	3	25	10
				
*Clinical characteristics*				
Neurological Impairment Score (NIS)				
cranial nerves	0	0	0	0
Muscle weakness	24	26	15	4
Reflexes	16	16	2	4
Sensation	24	6	6	2
**Total NIS score**	**64**	**48**	**23**	**10**
Charcot-Marie-Tooth disease neuropathy score (CMTNS)				
Sensory symptoms	3	0	3	3
Motor symptoms				
Legs	4	1	1	1
Arms	1	0	0	0
Pin sensibility	4	3	3	3
Vibration	4	4	3	-
Strength				
Legs	1	3	1	4
Arms	3	1	0	0
Ulnar/median CMAP	2	-	0	1
Ulnar/median SNAP	4	-	3	3
**Total CMTNS score**	**26**	**12**	**14**	**15**
Ataxia	Marked	Slight	-	-
Romberg	Positive	-	Positive	-
Pes cavus	Present	-	Present	Present
Kyphoscoliosis	Marked	Slight	0	0
				
*Phenotype*	Déjérine-Sottas syndrome	Charcot-Marie-Tooth type 2	Charcot-Marie-Tooth type 2	Charcot-Marie-Tooth type 2
				
*Missense mutation*	368G>A	103G>A	103G>A	103G>A
				
*Amino acid change*	Gly123Asp	Asp35Asn	Asp35Asn	Asp35Asn

## Discussion

Our main finding is the identification of two novel missense mutations in the *MPZ *gene. The phenotype/genotype correlation is likely in family 1, while three affected all carried the mutation in family 2 and 3. Unfortunately we did not have DNA from the unaffected family members. However, the literature describes other missense mutations in the *MPZ *gene that affects the same codons as our novel missense mutations [6,16-18]. Change of nucleotide 103 was not found in 100 unrelated controls, and change of nucleotide 368 was not found in 80 unrelated controls [16,18]. Thus, we suggest that the novel *MPZ *missense mutations are disease causing rather than polymorphisms.

### Phenotype

Missense mutations in the *MPZ *gene account for about 2% of patients with CMT type 1 [18]. The phenotype varies considerably from early to late age at onset. Clinically missense mutations in the *MPZ *gene can cause congenital hypomyelinating neuropathy (CH), DSS, CMT type 1, CMT type 2 and intermediate CMT [6,16-22]. The phenotypes CH and DSS are characterized by early onset and severe symptomatology, while the other phenotypes have a later age at onset and milder symptomatology. Our patients had phenotypes compatible with DSS and CMT type 2.

### Phenotype related to MPZ protein structure

Our patient with DSS had a Gly123Asp amino acid substitution. Another patient with DSS had a Gly123Cys, while a patient with CMT type 1 had a Gly123Ser amino acid substitution [6,18]. Details about the patient with CMT type 1 are unfortunately not available. Ser has a structure that is much more similar to Gly than both Asp and Cys. Thus, the Gly123Ser amino acid change may cause less conformational changes of P_0 _than the Gly123Asp and Gly123Cys amino acid change. Similarly, missense mutations in the codon that include residue number 124 can cause CMT type 1 or CMT type 2 [19,21,22]. This is probably due to the characteristics of the specific amino acid substitution and the conformational changes of the MPZ protein. Our patients with CMT type 2 had Asp35Asn amino acid substitution. Asp and Asn has very similar structure and probably only causes minor conformational changes of P_0_, even though Asp is negatively charged and Asn is polar. Asp35Tyr causes intermediate CMT, CMT type 1 and CMT type 2 [16,17]. Tyr, a polar amino acid, has a hydroxyphenyl group that replace Asp charged side chain. The conformational change is likely to be small.

HeLa cells tranfected with two late onset and two early onset MPZ mutations showed that the two late onset mutations had normal transportation of the P_0 _to the cell membrane and moderately reduced MPZ-mediated intercellular adhesion, while the two early onset mutations had different patterns [23]. One early onset type was correctly glycosylated and trafficked to the plasma membrane, but intercellular adhesion was strongly affected, while the other early onset type caused by deletion in the gene showed that the transcripted P_0 _was retained within the cytoplasm with secondary reduced adhesion. The Ser63del causes a demyelinating neuropathy in transgenic mice, similar to the Ser63del causes a CMT type 1 in humans [24-26]. Interestingly, the P_0 _Ser63del is retained in the endoplasmic reticulum, and it fails to be incorporated into myelin [24,25]. These are very interesting mechanisms which are not directly related to the conformational change of the P_0_. The retained P_0 _might be the mechanism that causes congenital hypomyelinating neuropathy (CH). The literature provides several examples on allelic heterogeneity, i.e. mutations in one gene can cause different phenotypes. An example is mutations in the CACNA1A gene, which causes familial hemiplegic migraine (FHM-1), episodic ataxia (EA-2) or spinocerebellar ataxia (SCA-6) [27,28]. FHM-1 is characterized by sensory and motor aura followed by headache. The FHM-1 families show both intra- and interfamilial variability. Affected from some FHM-1 families experience severe hemiplegic attacks, cerebral oedema and life threatening coma due to minor head trauma. The more common Charcot-Marie-Tooth genes identified so far show both locus and allelic heterogeneity. The allelic heterogeneity in the *MPZ *gene is characterized by different types of neuropathy, in contrast to different disorders caused by mutations in the CACNA1A gene. At present it is anticipated that four P_0 _proteins form a tetramer and link to a tetramer of the opposed membrane. To our knowledge expression of the *MPZ *alleles is unknown. If one allele is expressed per cell, it will either be a wild type or changed P_0_. However, if both alleles are expressed equally, tetramer assembly would statistically be in the order 1:16, 4:16, 6:16, 4:16, 1:16 for the different combinations of wild type and changed P_0_. Thus, only 1:256 (1:16^2^) will be normal tetramers that can link together. A sural nerve biopsy of a patient with CH caused by a mutation in P_0 _presented a characteristic picture of non-myelinated and poorly myelinated axons with basal lamina onion bulbs and lack of myelin breakdown products [29]. The histology supports that both alleles, i.e. the wild type and mutated allele, are expressed in each Schwann cell. If only one allele was expressed per cell, one would expect to observe both normal and abnormal Schwann cell histology, i.e. normal and abnormal myelination along the axon. The assembly of different tetramers may also explain the intrafamilial variation of symptoms and age at onset [16,24]. Even a concordant monozygotic twin pair was described with different progression, classified as Roussy-Lévy variant of CMT1 and CMT1, respectively [30]. We cannot exclude the importance of environmental factor with certainty. However we do not think the environment has a crucial role given proper nutrition and exclusion of other neuropathy inducing factors.

## Conclusions

We suggest that the phenotypic variation caused by different missense mutation in the *MPZ *gene is closely linked to the effects on the MPZ protein tetramers, which is crucial for axonal myelination.

## Competing interests

The authors declare that they have no competing interests.

## Authors' contributions

GJB acquired the material, conceived the study, participated in the design of the study and drafted the manuscript. JCS carried out the molecular genetic studies and the sequence alignment. MBR conceived the study, participated in the design of the study and drafted the manuscript. All authors read and approved the final manuscript.

## Supplementary Material

Additional file 1 **Table S2**. Neurophysiology in patients with Charcot-Marie-Tooth disease caused by point mutations in the *MPZ*. Click here for file
