# Supplementary material for: Two novel missense mutations in the myelin protein zero gene causes Charcot-Marie-Tooth type 2 and Déjérine-Sottas syndrome
Source: BMC Res Notes. 2010 Apr 12;3:99. doi: 10.1186/1756-0500-3-99 (PMC2861067; doi:10.1186/1756-0500-3-99)
Supplement: Additional file 1 — Table S2. Neurophysiology in patients with Charcot-Marie-Tooth disease caused by point mutations in the MPZ. [file 1756-0500-3-99-S1.DOC]

Table 2. Neurophysiology in patients with Charcot-Marie-Tooth disease caused by point mutations in the *MPZ* gene.

|  |  |  | |  |  | | | | | | | |  | | | | | |  |
| --- | --- | --- | --- | --- | --- | --- | --- | --- | --- | --- | --- | --- | --- | --- | --- | --- | --- | --- | --- |
|  | Sex | Age at | | R/L | Motor nerves | | | | | | | | Sensory nerves | | | | | | EMG  chronic  dener-  vation |
|  |  | Onset  (yrs) | Exami-nation  (yrs) |  | Median | | Ulnar | | Peroneal | | Tibial | | Median | | Ulnar | | Sural | |  |
|  |  |  |  |  | CMAP | CV | CMAP | CV | CMAP | CV | CMAP | CV | SNAP | CV | SNAP | CV | SNAP | CV |  |
| Normal values → | | |  |  | 4.0 | 49.0 | 4.0 | 49.0 | 3.0 | 41.0 | 3.0 | 41.0 | 12.0 | 46.0 | 17.0 | 47.0 | 17.0 | 44.0 |  |
|  | | |  |  |  |  |  |  |  |  |  |  |  |  |  |  |  |  |  |
| *Family 1* |  |  |  |  |  |  |  |  |  |  |  |  |  |  |  |  |  |  |  |
| Proband | ♀ | 2 | 7 | R | 4.0 | **6.7** | **-** | **-** | **0.2** | **-** | **0.1** | **-** | - | - | - | - | **-** | **23.1** | **Present** |
|  |  |  | 13 | R | **1.8** | **-** | **2** | **6.9** | **0.7** | **9.2** | **-** | **-** | **A** | **A** | - | - | **-** | **-** | **-** |
|  |  |  |  |  |  |  |  |  |  |  |  |  |  |  |  |  |  |  |  |
| *Family 2* |  |  |  |  |  |  |  |  |  |  |  |  |  |  |  |  |  |  |  |
| Father | ♂ | 70 | 73 | R | - | - | **-** | **-** | - | **31.0** | **-** | **-** | - | - | - | - | **-** | **-** | **Present** |
|  |  |  |  | L | - | - | **-** | **-** | **-** | **37.0** | **-** | **-** | **-** | - | - | - | **-** | **-** | **Present** |
| Proband | ♀ | 29 | 53 | R | 7.5 | **42.0** | 5.0 | 60.0 | **0.4** | **36.0** | **A** | **A** | **2.0** | **35.0** | **5.0** | 50.0 | **0.5** | **35.0** | **Present** |
|  |  |  |  | L | - | - | **-** | **-** | **0.8** | **37.0** | **0.5** | **40.0** |  |  |  |  | **2.0** | **34.0** | **Present** |
|  |  |  |  |  |  |  |  |  |  |  |  |  |  |  |  |  |  |  |  |
| *Family 3* |  |  |  |  |  |  |  |  |  |  |  |  |  |  |  |  |  |  |  |
| Proband | ♂ | 56 | 59 | R | - | - | **-** | **-** | **A** | **A** | **A** | **A** | - | - | - | - | **A** | **A** | **Present** |
|  |  |  |  | L | - | - | **-** | **-** | **A** | **A** | **A** | **A** | - | - | - | - | **1.0** | **-** | **Present** |
|  |  |  | 62 | R | 5.6 | **48.9** | 8.5 | 52.3 | **A** | **A** | **A** | **A** | **1.2** | **41.5** | **1.0** | **41.2** | **1.8** | **46.4** | **Present** |
|  |  |  |  | L | - | - | **-** | **-** | **-** | **-** | **-** | **-** | - | - | - | - | **0.4** | **42.7** | **-** |
|  |  |  | 63 | R | 4.4 | 49.0 | 7.7 | 55.0 | **A** | **A** | **A** | **A** | **1.4** | **48.0** | - | - | **A** | **A** | **Present** |
|  |  |  |  |  |  |  |  |  |  |  |  |  |  |  |  |  |  |  |  |

CMAP = compound motor action potential (mV); SNAP = sensory nerve action potential (µV); CV, conduction velocity (m/s); A,absent evoked response; -, not measured; R/L = right/left;

Bold = abnormal values
